# Cost-effectiveness of family history-based colorectal cancer screening in Australia

**DOI:** 10.1186/1471-2407-14-261

**Published:** 2014-04-16

**Authors:** Driss A Ouakrim, Alex Boussioutas, Trevor Lockett, John L Hopper, Mark A Jenkins

**Affiliations:** 1 Centre for Epidemiology and Biostatistics, The University of Melbourne, Melbourne, VIC 3010, Australia; 2 Peter MacCallum Cancer Centre, 3002 Melbourne, Australia; 3 Preventative Health national Research Flagship, CSIRO Food and Nutritional Sciences, North Ryde, NSW 2113, Australia

**Keywords:** Colorectal cancer, Family history, Screening, Cost-effectiveness

## Abstract

**Background:**

With 14.234 diagnoses and over 4047 deaths reported in 2007, colorectal cancer (CRC) is the second most common cancer and second most common cause of cancer-related mortality in Australia. The direct treatment cost has recently been estimated to be around AU$1.2 billion for the year 2011, which corresponds to a four-fold increase, compared the cost reported in 2001. Excluding CRCs due to known rare genetic disorders, 20% to 25% of all CRCs occur in a familial aggregation setting due to genetic variants or shared environmental risk factors that are yet to be characterised. A targeted screening strategy addressed to this segment of the population is a potentially valuable tool for reducing the overall burden of CRC.

**Methods:**

We developed a Markov model to assess the cost-effectiveness of three screening strategies offered to people at increased risk due to a strong family history of CRC. The model simulated the evolution of a cohort of 10,000 individuals from age 50 to 90 years. We compared screening with biennial iFOBT, five-yearly colonoscopy and ten-yearly colonoscopy versus the current strategy of the Australian National Bowel Cancer Screening Programme (i.e. base case).

**Results:**

Under the NBCSP scenario, 6,491 persons developed CRC with an average screening lifetime cost of AU$3,441 per person. In comparison, screening with biennial iFOBT, colonoscopy every ten years, and colonoscopy every five years reduced CRC incidence by 27%, 35% and 60%, and mortality by 15%, 26% and 46% respectively. All three screening strategies had a cost under AU$50,000 per life year gained, which is regarded as the upper limit of acceptable cost-effectiveness in the Australian health system. At AU$12,405 per life year gained and an average lifetime expectancy of 16.084 years, five-yearly colonoscopy screening was the most cost-effective strategy.

**Conclusion:**

The model demonstrates that intensive CRC screening strategies targeting people at increased risk would be cost-effective in the Australian context. Our findings provide evidence that substantial health benefits can be generated from risk-based CRC screening at a relatively modest incremental cost.

## Background

With 14.234 diagnoses and over 4047 deaths reported in 2007, colorectal cancer (CRC) is the second most common cancer and second most common cause of cancer-related mortality in Australia [1]. The direct treatment cost has recently been estimated to be around AU$1.2 billion for the year 2011, which corresponds to a four-fold increase compared the cost reported in 2001 [2,3].

Approximately 10-15% of the population have a family history of CRC, which increases the disease personal risk by two-four fold (excess familial risk) depending on the number of relatives affected, the degree of relationship of the affected relatives and the age of diagnosis [4]. Even after excluding CRCs due to known genetic disorders such as Lynch syndrome and familial adenomatous polyposis (FAP), the cause of about 25-50% of this excess familial risk is unknown [4,5] but could be due to specific genetic variants or shared environmental risk factors that are yet to be characterised [6].

The focus of this study is people at increased risk of CRC because of a strong family history of CRC. Here, we define strong family history as people with one first-degree relative diagnosed before the age of 55 years, or with two first-degree relatives or one first- and one second-degree relative on the same side of the family diagnosed at any age.

On average, these people have an estimated risk of developing CRC between three and six times higher compared to the average risk population [7,8].

A targeted screening strategy addressed to this segment of the population is a potentially effective approach to reduce the burden of CRC.

Whilst three randomised controlled trials have demonstrated that screening with faecal occults blood test (FOBT)—followed with a diagnostic colonoscopy in case of positive test—is effective in reducing CRC incidence and mortality in the average risk population, no direct evidence exists to support the effectiveness of screening in people in this increased risk category. Based on expert opinion, current guidelines generally advise that persons with a strong family history of CRC should initiate screening at an earlier age or undergo more intensive screening compared to the average risk population [9-11]. For example, current guidelines in the United States recommend colonoscopy screening every 10 years starting at age 40 or ten years younger than the age of the first diagnosis of CRC in the family [11,12] Similarly, the Australian National Health and Medical Research Council (NHMRC) guidelines designate persons in this risk category as being at “moderately increased-risk” of CRC and recommend colonoscopic screening every five years from the earlier of age 50 years or ten years younger than the age of the first diagnosis of CRC in the family [10].

Similar to the clinical effectiveness, the cost-effectiveness of CRC screening in the average-risk population, based on various strategies and using different screening modalities, has been established by several studies [13-18]. Pignone and colleagues identified six Australian studies published between 1996 and 2010. All of these studies concluded that annual and biennial CRC screening by guaiac-based or immunochemical faecal occult blood test (iFOBT) is cost-effective, with cost per life-year gained under $55,000 per year in 2010 Australian dollars [19]. Conversely, very few economic evaluation studies have focused on the economic aspects of screening in people at increased risk of CRC due to a strong family history, despite the fact that up to a quarter of all CRC cases are diagnosed in this segment of the population. We identified only two recent studies, conducted in Spain and the United States, assessing the economic implication of implementing a family history-based CRC screening programme [20,21]. Both studies found colonoscopic screening every five years starting at age 40 years to be a cost-effective strategy for people with a family history of CRC.

There is a strong rationale for designing risk-based CRC screening policies (i.e. intensiveness of screening based on risk) as this would, theoretically at least, permit a more efficient allocation of limited health care resources [22]. However, such policies, based on a personalised approach of CRC screening, would also have an important economic impact on the health care system as a whole (e.g. familial risk assessment programmes, higher number of screening colonoscopies and more specialists able to perform them) and overall public health implications which need to be investigated.

The main objective of this analysis was to provide an economic evaluation for people at increased risk of CRC, based on Australian data assessing the cost-effectiveness of biennial iFOBT, five-yearly colonoscopic screening starting at age 50 years, and ten-yearly colonoscopic screening starting at age 50 years to the current program of the National Bowel Cancer Screening programme of Australia (NBCSP) which currently consists of a one-off iFOBT screening offered at age 50, 55, 60 and 65 years (9) irrespective of family history.

## Methods

**Figure 1:**
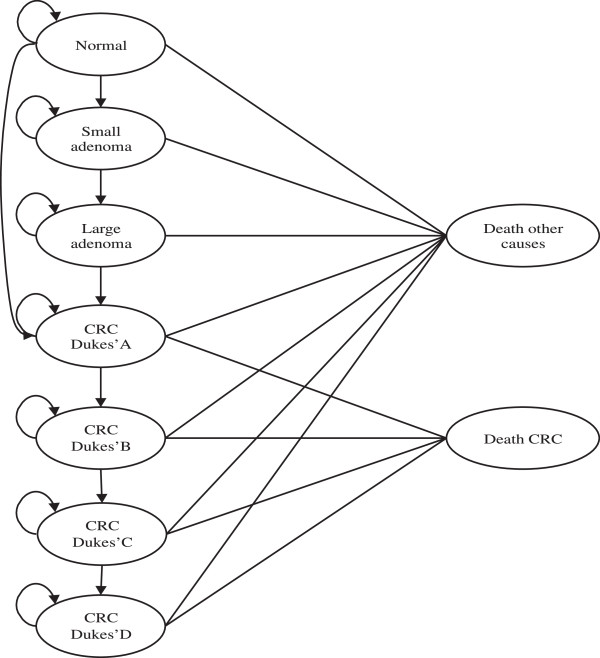
**Markov process for disease natural history and colorectal cancer diagnosis.** Persons in the modelled cohort go through this cycle every year from age 50 to 90.

We developed a Markov microsimulation model to simulate the natural history of CRC and to evaluate costs and outcomes of screening. Figure 1 summarises the model structure and assumptions. Four CRC screening strategies were superimposed on this model to simulate the evolution of a hypothetical population of 10,000 individuals at “moderately increased risk” of CRC due to a strong family history-as defined by the current NHMRC guidelines [10].

Based on current understanding of the variability in the natural history of the disease, we created nine mutually exclusive, possible health states (from normal to death due to CRC or other causes) and specified transition probabilities for movements between these health states. An individual can remain in the same health state or move to another health state according to the predetermined transition probabilities. Costs and health outcomes (including life expectancy) were assigned to each state and transition. The software TreeAge Pro was used to implement the model [23].

### Model parameters

The parameters used in the Markov model are presented in Table 1. All individuals in the simulated cohort entered the model at age 50 years and were able to exit the model only when they turned 90 years of age or via one of the two absorbing states. Participants’ initial state was distributed to normal, adenoma or CRC according the corresponding prevalences at age 50 years based on assumptions provided below (Table 2). The model assumed that around 85% of all CRCs developed from large adenomas, which developed from small adenomas, which arose from normal bowel.

**Table 1:** Parameters of the model

**Variable**	**Value**	**Reference**
**Natural history**		
**Age-specific incidence Adenoma <10 mm**		
50-54	0.24	Bishop et al. (derived from prevalence and incidence data presented in tables 32 and 35)
55-59	0.30	
60-64	0.32	
65-69	0.27	
≥ 70	0.22	
**Age-specific incidence Adenoma ≥10 mm**		
50-54	0.07	
55-59	0.08	
60-64	0.1	
65-69	0.16	
≥ 70	0.21	
**CRC Dukes’ A**		
50-54	0.0012	
55-59	0.0022	
60-64	0.0033	
65-69	0.005	
≥ 70	0.0067	
**CRC Dukes’ B**		
50-54	0.0005	
55-59	0.001	
60-64	0.0016	
65-69	0.0023	
≥ 70	0.0031	
**CRC Dukes’ C**		
50-54	0.0005	
55-59	0.0008	
60-64	0.0013	
65-69	0.002	
≥ 70	0.0027	
**CRC Dukes’ D**		
50-54	0.0001	
55-59	0.0003	
60-64	0.0004	
65-69	0.0007	
≥ 70	0.0009	
**Yearly probability of death from CRC by stage**		
Dukes’ A	0.034	
Dukes’ B	0.051	
Dukes’ C	0.085	Tran et al.
Dukes’ D	0.282	
**Probability of CRC diagnosis without screening programme**		
Dukes’ A	0.091	Bishop et al. (table 34)
Dukes’ B	0.2948	
Dukes’ C	0.7613	
Dukes’ D	1	
**Screening test characteristics**		
**iFOBT**		
Sensitivity for CRC	0.479	Bishop et al. (table 36)
Sensitivity for polyps’	0.2119	Bishop et al. (table 36)
Specificity	0.9146	Bishop et al. (table 36)
Screening uptake	40%	NBCSP pilot
Probability of colonoscopy after positive iFOBT	65%	
**Colonoscopy**		
Sensitivity for CRC	0.95	Bishop et al. (table 36)
Sensitivity for polyps	0.85	NHMRC 2005
Specificity	1	Bishop et al. (table 36)
Participation	40%	Assumption
**Cost per activity per individual (AU$)**		
iFOBT (invitation + test kit)	$10.00	Bishop et al. (table 40)
iFOBT pathology	$20.00	
GP visit and referral	$32.10	
Colonoscopy	$1,082.00	
Colonoscopy with polypectomy	$1,606.00	
**Annual CRC treatment costs per individual (AU$)**		
CRC Dukes’ A	$1,716.00	Tran et al.
CRC Dukes’ B	$4,114.00	
CRC Dukes’ C	$9,990.50	
CRC Dukes’ D	$35,578.00	

**Table 2:** Possible states for cohort to enter the model

**State**	**Description**	Probability*
Normal	No abnormality	0.78
Adenoma <10 mm	Individual has adenoma that is <10 mm in diameter	0.1668
Adenoma ≥10 mm	Individual has adenoma that is ≥10 mm in diameter	0.0464
Surveillance	Individuals are in the surveillance state once they have had an adenoma ≥ 10 mm removed	0
CRC Dukes’ A	Individual has developed CRC and is at Dukes stage A	0.0036
Treatment Dukes’ A	Individual with Dukes stage A has been diagnosed and will not take part in any further screening	0
CRC Dukes’ B	Individual has developed CRC and is at Dukes stage B	0.0016
Treatment Dukes’ B	Individual with Dukes stage B has been diagnosed and will not take part in any further screening	0
CRC Dukes’ C	Individual has developed CRC and is at Dukes stage C	0.0012
Treatment Dukes’ C	Individual with Dukes stage C has been diagnosed and will not take part in any further screening	0
CRC Dukes’ D	Individual has developed CRC and is at Dukes stage D	0.0004
Treatment Dukes’ D	Individual with Dukes stage D has been diagnosed and will not take part in any further screening	0
Dead CRC	Death from CRC	0
Dead other causes	Death from colonoscopy	0

*Based on Bishop et al.’s prevalence estimates (table 32) [24]. The original figures were multiplied by a factor of 4 for this analysis (methods section).

Age-specific incidence rates of small polyps, large polyps, adenomas and CRC at different stages were obtained from the health economics review conducted in 2008 by Bishop et al. for the Cancer Institute of New South Wales [24].

To reflect a cohort of persons in the “moderately increased risk” category (defined as: one 1st degree relative with CRC diagnosed before age 55 years; or two 1st or one 1st and one 2nd degree relative/s on the same side of the family diagnosed at any age), we followed the approach adopted by Ladabaum et al. [20] and Ramsey et al. [21]. We multiplied the population’s prevalence of adenoma and CRC at age 50 by a factor of four (relative risk (RR) = 4) on the basis of the current NHMRC criteria of 3 to 6 fold increased risk in this population compared to the average risk population [10]. We similarly, multiplied the estimates of incidence of small polyps, large polyps and CRC at different stages for the general population by four to calculate the age-specific transition probabilities and determine the participants’ progression through the different cycles of the model. In the sensitivity analysis, we varied the RR between 3 and 6. Age-specific probabilities of death due to causes other than CRC were obtained from life tables published by the Australian Institute of Health and Welfare [25]. Stage-specific annual CRC mortality rates were based on recent five-year survival data derived from the BioGrid Australia dataset and presented by Tran and colleagues [3].

### Screening programmes

We compared four alternative CRC screening strategies:

1. Screening according to the current NBCSP programme. Under this screening scenario, which was used as the baseline strategy in the model, participants were invited to undertake CRC screening with an immunochemical iFOBT at age 50, 55, 60 and 65 years (Usual Care).

2. iFOBT biennial screening (every two years) from age 50 years (iFOBT_2_).

3. Colonoscopy screening every five years starting at age 50 years. This strategy reflects the current screening recommendation for people at moderately increased risk of CRC (COLO_5_).

4. Colonoscopy screening every ten years starting at age 50 years (COLO_10_).

In both iFOBT strategies (Usual Care and iFOBT_2_), a person invited to screen could “choose” to undertake (screeners) or to decline screening (non-screeners). We assigned screener status on a 40% probability based on the participation rate observed in the NBCSP reported in the latest monitoring report of the programme [26].

The proportion of screeners with positive (faecal occult blood detected) or negative tests (no faecal occult blood detected by FOBT) was determined by the participant’s probability of disease status (i.e. presence of adenoma or CRC) and the diagnostic accuracy of the test (i.e. sensitivity and specificity). Participants with negative tests were re-invited to screen according to the protocol of each iFOBT-based screening strategy. We assumed that all screeners with a positive iFOBT result were eligible for a follow-up diagnostic colonoscopy referred by a general practitioner. The compliance for the recommended diagnostic procedure was also based on the compliance observed in the NBCSP. If diagnostic colonoscopy confirmed the presence of a small adenoma (<10 mm), a polypectomy was assumed to having been performed and the participant returned to the normal state in the natural history model. Subjects with large adenomas (≥10 mm) exited the screening programme after treatment and were followed up with five-yearly surveillance colonoscopy as recommended in the current NHMRC guidelines [10].

For colonoscopy-based screening strategies, a similar logic to that of iFOBT-based screening was applied, the only differences being the direct removal of any detected polyp and the use of colonoscopy values for sensitivity and specificity. In the base case model, level of screening uptake was determined based on a meta-analysis of eight studies reporting colonoscopy screening participation rates for people at increased risk of CRC due to family history [27]. Subjects with a CRC diagnosed through screening entered a treatment state, according to their CRC stage, and progressed through increasing disease stages until the end of the Markov process. They were able to exit the model only via death due to CRC or other causes. Subjects who decline CRC screening, those with false negative results (existing faecal blood, polyp or CRC tumour undetected) and those who decline diagnostic colonoscopy after a positive iFOBT returned to the disease natural history.

### Costs

For this analysis we adopted a third-party payer (health care system) perspective. Only direct costs, such as unit cost or annual costs related to screening and diagnostic tests, adenoma surveillance and CRC treatment were taken into account. Indirect costs related to production loss due to undergoing screening, colonoscopy or hospitalisation, were not considered in this analysis. Screening and diagnostic costs were based on the estimates reported by Bishop et al. [24]. For iFOBT based screening, costs included those needed for the organisation of the screening campaign (public information, programme development, infrastructure and coordination) and purchasing, distributing and interpreting the tests (pathology processing and information costs) as well as the diagnostic follow up of positive iFOBTs. For colonoscopy based screening strategies, we assumed the same programme organisation and colonoscopy test costs as for the FOBT screening strategies.

Annual costs of treating CRC according to stage at diagnosis were derived from lifetime treatment costs: AU$34,337 for treating a Dukes’ A CRC, AU$53,487 for Dukes’ B, AU$79,924 for Dukes’ C and AU$71,156 for Dukes’ D, and represent the average cost to the Australian health system for an individual diagnosed with CRC over the course of their life [3]. To calculate predicted costs and outcomes in terms of their present value, all future costs and outcomes were discounted at an annual rate of five percent (the standard discount rate currently used by Australian health technology assessment agencies such as the Pharmaceutical Benefits Scheme). This reflects the advantage an individual gets from receiving a benefit earlier or incurring a cost later in time and is known as time preference [28]. Here, the discount rate adjusts for policy maker’s time preference for present over future outcomes in terms of the costs and effects resulting from each screening strategy. The performance of each screening strategy was measured using the incremental cost-effectiveness ratio (ICER). This indicator is defined as the cost for each unit increase in effect and is obtained by calculating the difference in cost between two alternative screening strategies divided by the difference in effect between the same strategies [29]. We also determined the total number of CRC cases by stage, and average life years gained (LYG) and costs per person. Sensitivity analyses were conducted to assess the impact of different screening participation rates and CRC incidence risk on the model’s results. To relate colonoscopy utilisation to the number of lives saved, the total number of procedures performed was divided by the total CRC deaths avoided in each screening scenario compared with the NBCSP.

## Results

Table 3 presents the results of the base-case model. Under the Usual Care screening scenario (one-off iFOBT screening offered at age 50, 55, 60 and 65), 6,491 out of a cohort of 10,000 “moderately increased-risk” 50 year-old people would develop CRC over 40 years (to age 90) with an average lifetime cost of AU$3,441 per person. Compared to the NBCSP, the iFOBT_2_, COLO_10_ and COLO_5_ screening strategies reduced the number of CRC cases by 27%, 35% and 60% and CRC mortality by 15%, 26% and 46% respectively. All three approaches resulted in more LYG and were more costly than Usual Care. Five-yearly colonoscopy screening starting at age 50 was the most costly option with a lifetime cost estimated to be AU$8,734 per person but also provided the highest LYG of all the screening strategies.

**Table 3:** Number of expected clinical events for each screening strategy in the 40-year follow up model

	**NBCSP screening**	**iFOBT every two years**	**Colonoscopy every 5 years**	**Colonoscopy every 10 years**
**CRC cases occurring per 10,000 persons from age 50 to 90 years**	6,491	4,745	2,568	4,205
**Reduction in CRC incidence compared with NBCSP**		27%	60%	35%
**CRC by stage**				
Dukes’ A	3,749	3,513	1,974	3,177
Dukes’ B	1,182	783	380	660
Dukes’ C	424	323	165	289
Dukes’ D	153	126	49	79
**Deaths attributable to CRC**	983	830	524	722
**Reduction in CRC mortality compared with NBCSP**		15%	46%	26%
**Total Number of colonoscopies**	21,333	72,885	167,031	109,213
**Number of colonoscopies per life saved**		476	363	418

iFOBT_2_: immunochemical faecal occult blood test, COLO_5_: colonoscopy screening every five years, COLO_10_: colonoscopy screening every ten years.

**Figure 2:**
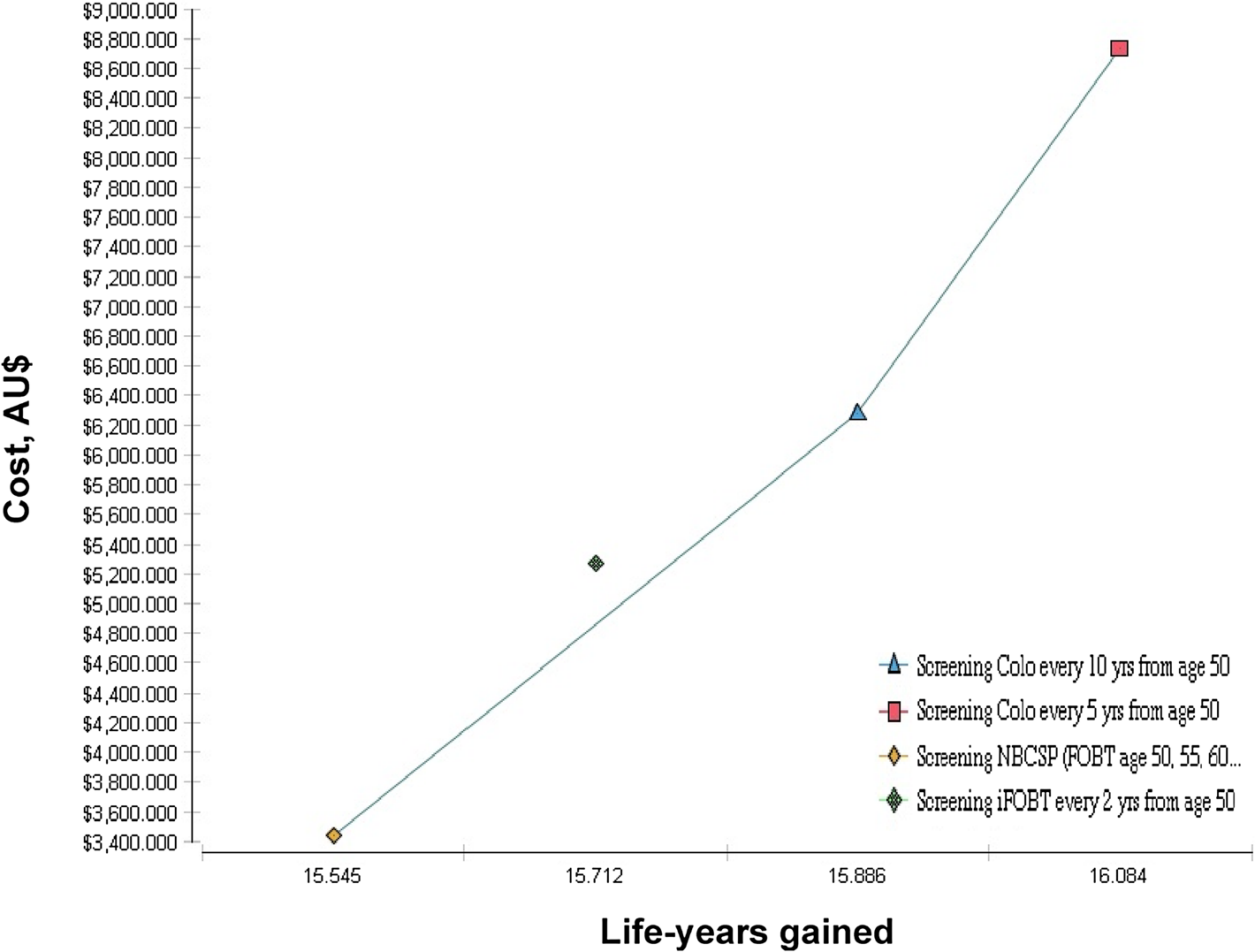
Cost-effectiveness of the screening strategies included in the model.

The incremental cost-effectiveness ratios of the different screening strategies compared to Usual Care are shown in Table 4. These ratios were AU$8,306 and AU$12,405 per LYG for COLO_10_ and COLO_5_ respectively. iFOBT_2_ was dominated by both screening approaches in the model (Figure 2). The COLO_10_ strategy had the lowest ICER compared to usual care. However, with a superior clinical effect (longer life expectancy and 60% reduction of incident CRC cases over the 40-year period) and the lowest number of colonoscopy procedures needed to save one life, COLO_5_ screening appeared as the most cost-effective strategy of the models compared.

**Table 4:** Incremental cost-effectiveness ratios for alternative CRC screening strategies compared current NBCSP

**Screening strategy**	**Total average lifetime costs (AU$)**	**Incremental costs (AU$)**	**Total average lifetime expectancy (years)**	**ICER (AU$/LYG)**
**NBCSP screening (base case)**	3,441		15.545	
**Colonoscopy every 10 years**	6,278	2,837	15.886	8,306
**Colonoscopy every 5 years**	8,734	2,456	16.084	12,405

ICER: incremental cost-effectiveness ratio.

Sensitivity analyses showed that above 20% participation rate the level of colonoscopy screening uptake was not a very influential parameter on the ICER (Table 5). For example, in a direct comparison with the NBCSP scenario, with a 20% screening participation rate, the ICERs for the COLO_5_ and COLO_10_ strategies were $9,108 and $7,835 per LYG respectively. The cost of both strategies were positively correlated with the level of screening uptake but increased by only 13% and 24% respectively when screening participation was set to 90%.

**Table 5:** One-way sensitivity analysis of ICER based on screening participation rate

**Colonoscopy screening participation rate**	**ICER COLO**_**5 **_**vs. usual care**		**ICER COLO**_**10 **_**vs. usual care**	
	**Life years gained per 1,000 persons**	**ICER (AU$)**	**Life years gained per 1,000 persons**	**ICER (AU$)**
10%	81	17,779	-	Dominated
20%	346	9,108	148	7,835
30%	492	9,089	272	7,612
40% (reference)	561	9,842	361	8,619
50%	699	9,283	416	9,192
60%	717	10,095	476	9,863
70%	775	10,245	547	9,887
80%	824	10,279	609	9,949
90%	871	10,423	641	10,333
100%	880	11,139	722	10,384

ICER: incremental cost-effectiveness ratio, iFOBT_2_: immunochemical faecal occult blood test, COLO_5_: colonoscopy screening every five years, COLO_10_: colonoscopy screening every ten years.

Effects of varying the level of CRC risk (i.e. 3 and 6 fold risk increase compared to 4 fold increase used in the base-case model) associated with a strong family history on the different outcomes of the model are presented in Additional file 1: Table S1-S4.

## Discussion and conclusions

Our aim was to provide an insight into the cost-effectiveness of CRC screening for people at increased risk of the disease due to having a strong family history. We compared three different screening alternatives to the current NBCSP using Australian data on CRC incidence, adenoma distribution and cancer stage for invasive carcinoma. We chose the current NBCSP screening strategy as the base case scenario in our model instead of a no screening scenario as we consider this approach to be more realistic and more relevant to the Australian context where a population-based screening programme has been in place since 2006 and will be expended to cover all individuals aged 50 to 75 years by 2017 [30].

All three strategies had an ICER under $50,000 per LYG, which is regarded as the upper limit of acceptable cost-effectiveness in the Australian health system [24,31]. An incremental cost of AU$12,405 per LYG, meant colonoscopy screening every five-year appears to be the most cost effective strategy of the three tested. In comparison, the Australian study conducted by Tran and colleagues (from which we obtained the CRC survival rates and treatment costs for our modelling) found the NBCSP, as implemented in 2008, to be cost-effective for the general population at AU$38,216 per LYG [3].

Ladabaum et al. conducted a cost-effectiveness analysis including only direct medical costs and using data from a screening pilot programme implemented in Aragón (Spain) targeting first-degree relatives of patients with CRC. The authors analysed the cost-effectiveness of CRC screening starting at age 40 years comparing colonoscopy every ten years and colonoscopy every five years, to no screening. Both screening strategies dominated the “no screening” option. Similar to our findings, five-yearly colonoscopic screening was found to be the most cost-effective strategy [20].

In a study conducted in the United States, Ramsey and colleagues used a validated microsimulation model [32] adopting a societal perspective (i.e. analysis including direct and indirect costs of screening) to evaluate the clinical and economic implications of implementing a CRC screening programme based on family history in the United States. Similar to our approach, the authors used a very specific definition of family history, based on current CRC screening guidelines. In the model, persons with a positive family history (i.e. having one FDR diagnosed with CRC before age 60 or two FDRs diagnosed at any age) had three alternative colonoscopy-based screening scenarios, which varied by frequencies (five or ten years) and age at which the screening began (age 40 to age 50). Results from each one of these screening strategies were then compared to a “usual care” scenario where colonoscopic screening was offered every ten years to the entire population (average- and increased-risk persons) from age 50. The analysis found all screening strategies to have acceptable ICERs (including the usual care option when compared to no screening) with five-yearly colonoscopy screening from age 40 years being the most cost-effective option (US$18,678 per LYG). The study concluded that a combination of a more aggressive CRC screening strategy targeting people with a family history, with a programme addressed to the general population may be a valuable approach to prevent CRC in the population [21].

In this study we attempted to conduct a similar analysis in the Australian context where an iFOBT-based screening programme has been in place since 2006. To our knowledge, this is the first analysis attempting to measure the economic aspect of different CRC screening strategies (including NHMRC recommendations) that target persons at “moderately increased risk” of CRC in the Australian context. It should be noted that this category does not include all individuals with a family history of CRC. Our model is particularly relevant to a segment of the population at a substantially increased risk of CRC—namely, individual whom CRC family history characteristics place them in the risk category 2 of the current Australian guidelines [10]. Persons in this category are more vulnerable to CRC due to their familial risk but also due to the fact that the current NBCSP strategy, by definition, does not address their specific needs in terms of screening modality and intensity. We believe that there is an urgent need to adjust the existing screening programme in order to address the specific screening requirements for this population. Our estimates of the number of colonoscopies needed for each screening scenario represents a proxy for resource utilization as well as adverse events from screening. Currently, over 500,000 colonoscopies are performed each year in Australia [33], a substantial proportion of these procedures occur outside of the national screening programme [19] and are undertaken by individuals at average risk of CRC for whom colonoscopy is not recommended as a screening procedure [34]. In this context, adjusting the current NBCSP to appropriately address the screening needs of people above average risk of CRC is likely to mainly consist of a more efficient use of already existing resources.

The modelling presented here has several limitations. For example the model assumed that family history of CRC was known for all participants and did not include administrative costs associated with the implementation of a family history assessment.

While the model accounted for the higher incidence of polyps observed in people with family history of CRC as a cause of a higher CRC incidence [35], it did not include information on polyp sojourn time (i.e. preclinical phase), which determines the rate of cancerous transformation. Polyp behaviour is those with family history is still not well characterised, particularly the malignancy transformation rate.

Our analysis was conducted from a third-party payer perspective and was limited to direct costs only. Indirect costs such as production loss due to CRC treatment need be taken into account for a more exhaustive economic assessment of a family history-based CRC screening programme. We did not take into account the costs involved with potential complications from screening tests, and the combination of screening and administrative costs. Also, our costs parameters rely heavily on estimates reported in Bishop et al. review, which was published in 2008. It is possible that some of those estimates might be out-of-date given the constant progress and changes in CRC treatment protocols.

In conclusion, our results are consistent with findings from previous research and present informative preliminary estimates in the perspective of familial risk-based CRC screening strategies within the context of a national screening programme addressed to the average risk population. Given limited colonoscopy capacity and budget, there is an increasing number of investigators calling for a better inclusion of personal disease risk in the design of CRC screening policies. The rational of this approach being that more screening resources should be allocated to those who would benefit most from more screening while at the same time reducing the intensity of screening among those who have less to gain. Several risk prediction tools have been developed [36,37] and future advances in genetic testing for CRC variants will facilitate this process, to allow tailored screening strategies. Our findings suggest that health benefits can be generated by implementing more intensive screening to those at increased risk at a relatively modest incremental cost.

## Competing interests

The authors have no competing interests to declare in relation to this manuscript.

## Authors’ contributions

DA designed the study, carried out data collection, statistical analysis and writing of the manuscript. MJ participated in the design of the study, the interpretation of the statistical analysis and the writing of the manuscript. TL, AB and JH participated in the design of the study and helped to draft the manuscript. All authors read and approved the final manuscript.

## Pre-publication history

The pre-publication history for this paper can be accessed here:

http://www.biomedcentral.com/1471-2407/14/261/prepub

## Supplementary Material

Additional file 1: Table S1-S4 Description of data: Results of cost-effectiveness analysis with three and six fold increased risk of colorectal cancer. Click here for file
