# Supplementary material for: Cost-effectiveness of family history-based colorectal cancer screening in Australia
Source: BMC Cancer. 2014 Apr 16;14:261. doi: 10.1186/1471-2407-14-261 (PMC4021190; doi:10.1186/1471-2407-14-261)
Supplement: Additional file 1: Table S1-S4 — Description of data: Results of cost-effectiveness analysis with three and six fold increased risk of colorectal cancer. [file 1471-2407-14-261-S1.docx]

**Additional file 1: Table S1:** Clinical results for model with individuals at three times higher risk of colorectal cancer

|  | **NBCSP screening** | **iFOBT every two years** | **Colonoscopy every 5 years** | **Colonoscopy every 10 years** |
| --- | --- | --- | --- | --- |
| **CRC cases per 10,000 persons form age 50 to 90** | 5,098 | 3,498 | 2,555 | 3,215 |
| **Reduction in CRC incidence compared with NBCSP** |  | 31% | 50% | 37% |
| **CRC by stage** |  |  |  |  |
| Dukes' A | 3,931 | 2,605 | 2081 | 2,453 |
| Dukes' B | 790 | 572 | 324 | 543 |
| Dukes' C | 300 | 244 | 114 | 176 |
| Dukes' D | 77 | 77 | 36 | 43 |
|  |  |  |  |  |
| **Deaths attributable to CRC** | 705 | 621 | 437 | 474 |
| **Reduction in CRC mortality compared with NBCSP** |  | 12% | 38% | 33% |
| **Total Number of colonoscopies** | 18,160 | 64,792 | 145,251 | 95,427 |
| **Number of colonoscopies per life saved** |  | 771 | 542 | 413 |

**Additional file 1: Table S2.** Incremental cost-effectiveness ratios for alternative CRC screening strategies compared to the existing NBCSP for individuals at three times higher risk of CRC

| Screening strategy | **Total average lifetime costs (AU$)** | **Incremental costs (AU$)** | **Total average lifetime expectancy (years)** | **ICER (AU$/LYG)** |
| --- | --- | --- | --- | --- |
| NBCSP screening | 2,557 | -- | 15.562 | -- |
| Colonoscopy every 10 yrs | 5,288 | 2,731 | 15.821 | 10,581 |
| Colonoscopy every 5 yrs | 7,688 | 2,399 | 16.001 | 13,270 |

ICER: incremental cost-effectiveness ratio

**Additional file 1: Table S3** Clinical results for model with individuals at six times higher risk of CRC

|  | **NBCSP screening** | **iFOBT every two years** | **Colonoscopy every 5 years** | **Colonoscopy every 10 years** |
| --- | --- | --- | --- | --- |
| **CRC cases per 10,000 persons form age 50 to 90** | 9,386 | 5,766 | 3,832 | 5,774 |
| **Reduction in CRC incidence compared with NBCSP** |  | 38% | 59% | 38% |
| **CRC by stage** |  |  |  |  |
| Dukes' A |  |  |  |  |
| Dukes' B | 1,746 | 1,164 | 607 | 1,054 |
| Dukes' C | 652 | 510 | 239 | 386 |
| Dukes' D | 186 | 150 | 75 | 119 |
|  |  |  |  |  |
| **Deaths attributable to CRC** | 1,388 | 1,189 | 721 | 1,045 |
|  |  | 199 | 667 | 343 |
| **Reduction in CRC mortality compared with NBCSP** |  | 14% | 48% | 25% |
| **Total Number of colonoscopies** | 21,962 | 75,546 | 165,284 | 107,094 |
| **Number of colonoscopies per life saved** |  | 379 | 248 | 312 |

**Additional file 1: Table S4.** Incremental cost-effectiveness ratios for alternative CRC screening strategies compared to the existing NBCSP for individuals at six times higher risk of CRC

| Screening strategy | Total average lifetime costs (AU$) | Incremental costs (AU$) | Total average lifetime expectancy (years) | ICER (AU$/LYG) |
| --- | --- | --- | --- | --- |
| NBCSP screening | 4,855 | -- | 15.397 | -- |
| iFOBT every two years | 6,981 | 2,126 | 15.563 | 12,795 |
| Colonoscopy every 5 yrs | 10,527 | 3,546 | 15.826 | 13,511 |

ICER: incremental cost-effectiveness ratio
